# Urogenital Cancer Epidemiology in Poland (1980–2020): A Narrative Review

**DOI:** 10.3390/cancers17020316

**Published:** 2025-01-20

**Authors:** Iwona Wnętrzak, Joanna Didkowska, Roman Sosnowski, Mateusz Czajkowski, Dawid Sigorski, Bartosz Małkiewicz, Piotr Marczyński, Jarosław Jaskulski, Piotr Kania, Adam Ostrowski, Artur Sieczych, Grzegorz Kade, Piotr Purpurowicz, Stanisław Szempliński, Robert Nowakowski

**Affiliations:** 1 Department of General and Oncological Urology, Praski Hospital, 03-401 Warsaw, Poland; 2 Department of Epidemiology and Cancer Prevention, Polish National Cancer Registry, Maria Sklodowska-Curie National Research Institute of Oncology, 02-781 Warsaw, Poland; 3 Department of Urology and Oncological Urology, MSWiA Hospital, Warmian-Masurian Cancer Center, 10-228 Olsztyn, Poland; 4 Department of Urology, Medical University of Gdańsk, Mariana Smoluchowskiego 17 Street, 80-214 Gdańsk, Poland; 5 Department of Oncology, University of Warmia and Mazury in Olsztyn, 10-228 Olsztyn, Poland; 6 Department of Minimally Invasive and Robotic Urology, University Center of Excellence in Urology, Wroclaw Medical University, 50-367 Wrocław, Poland; bmalkiew01@gmail.com; 7 Department of Urology, Holycross Cancer Center, 25-734 Kielce, Poland; 8 Department of Urology and Oncological Urology, St. John Paul II Mazovian Regional Hospital in Siedlce, 08-110 Siedlce, Poland; 9 Department of Urology and Andrology, Collegium Medicum in Bydgoszcz, Nicolaus Copernicus University in Torun, 85-067 Bydgoszcz, Poland; 10 Military Institute of Aviation Medicine, 01-755 Warsaw, Poland; 11 The Municipal Polyclinical Hospital in Olsztyn, 10-045 Olsztyn, Poland; 12 Second Department of Urology, Centre of Postgraduate Medical Education, 01-809 Warsaw, Poland

**Keywords:** epidemiology, urogenital cancer, testicular cancer, penile cancer, prostate cancer, bladder cancer, kidney cancer

## Abstract

Despite advances in prophylaxis, early diagnosis, and treatment, urogenital cancers represent a significant challenge to public health in Poland due to their relatively high prevalence and mortality rates. This narrative review aims to explore contemporary evidence on the epidemiology of urogenital cancers in Poland, such as prostate cancer, bladder cancer, kidney cancer, testicular cancer, and penile cancer, focusing on current and historical status and trends in the broader context of healthcare delivery. The literature consistently indicates that urogenital cancer continues to be a significant contributor to cancer incidence and mortality rates in Poland. Although the body of evidence is expanding, its quantity remains limited, primarily attributable to the scarcity of top-notch epidemiological investigations targeting particular forms of cancer, such as testicular and penile cancers, which are characterized by sporadic occurrences.

## Simple Summary

Malignant diseases represent the second leading cause of excess mortality among women and men in Poland. Urogenital cancer epidemiology in Poland is a seldom researched topic in the literature. In our work, we presented reliable data on this subject based on published work from the PubMed and Google Scholar databases, data from the Polish National Cancer Registry, and data from Statistics Poland. We discussed prostate cancer, bladder cancer, kidney cancer, testicular cancer, and penile cancer. Cancer registration in PNCR is mandatory, which ensures a high level of completeness of the data. Some data also come from the National Health Fund. We analyzed mortality, morbidity and survival. We discussed the risk factors, including Poland. We presented the limitations of the time, quantity, and sources of the existing studies, and emphasized the urgency of updating the research.

## 1. Introduction

Cancer epidemiology is of particular importance [1] because malignant diseases represent the second leading cause of excess mortality among women and men in Poland [2].

The term urogenital cancer used in this work covers two areas: cancers of the urinary system, occurring in both sexes (bladder cancer, kidney cancer), and cancers of the reproductive system, occurring in men (prostate cancer, testicular cancer, penile cancer). Both of these areas in the Polish health care system are dealt with by urologists.

Regarding urogenital cancer, the contribution of prostate, bladder, and kidney cancers to total disability-adjusted life years (DALY) in Poland was 4.3% and 3%, respectively [2].

**Figure 1:**
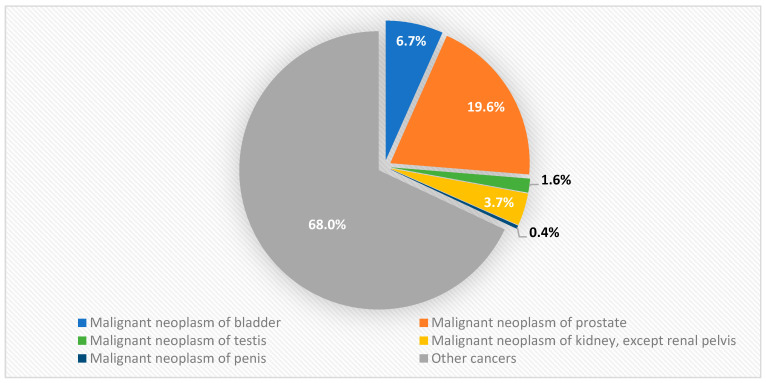
Male urogenital cancer incidence in Poland in 2020.

**Figure 2:**
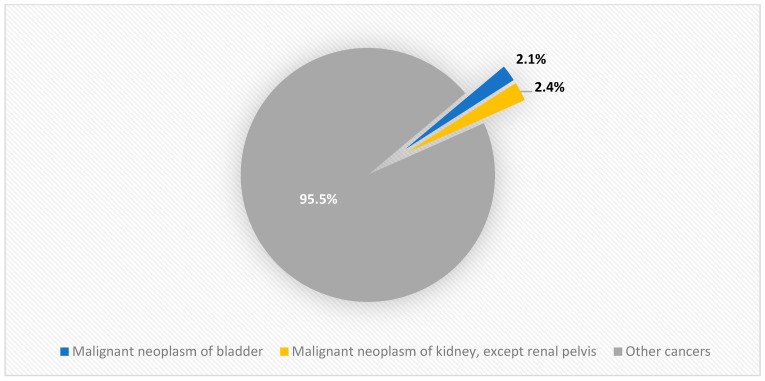
Female urinary tract cancer incidence in Poland in 2020.

**Figure 3:**
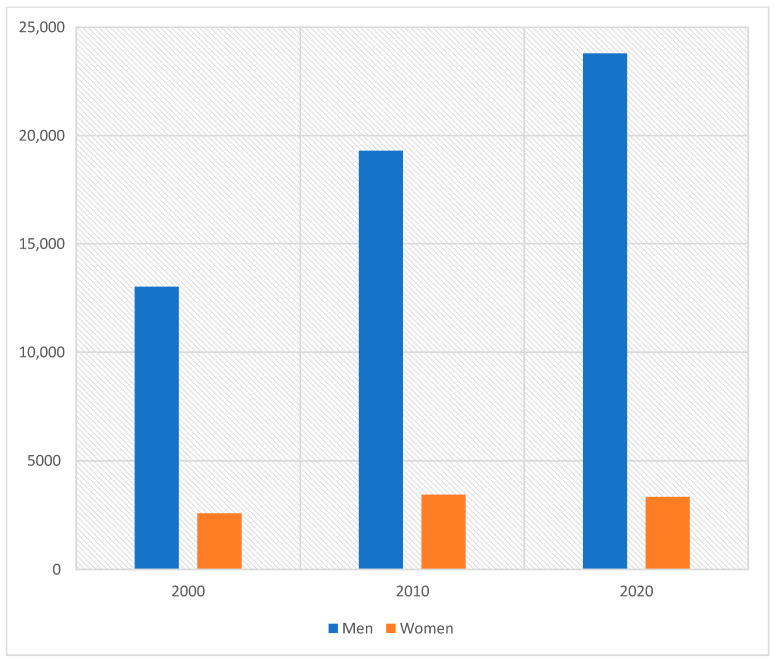
Urinary tract and genital cancer incidence among women and men in Poland in 2000, 2010, and 2020.

According to the Polish National Cancer Registry (PNCR), in 2020, urogenital cancers accounted for approximately 33% of male cancer incidence (about 37,761 new cases in 2020) and less than 5% of female cancer incidence (about 3466 cases). Figure 1 shows the occurrence of male urogenital cancer in Poland in 2020, while Figure 2 illustrates the occurrence of female urinary tract cancer in the same year. Furthermore, Figure 3 presents the incidence of urinary tract and genital cancer in women and men in Poland in 2000, 2010, and 2020.

Regarding mortality, urogenital cancers accounted for approximately 20% of cancer-related deaths among Polish males (16,560 cases) and approximately 4% among females (3466 cases) [3].

In 2020, an isolated incidence drop was observed due to the Coronavirus disease 2019 (COVID-19) pandemic, which resulted in 16.6% and 15.7% decreases in new cancer cases reported to the PNCR in men and women, respectively [3].

The data reported in the 2020 PNCR indicate that prostate cancer ranks first in terms of cancer incidence among men, with a contribution of 19.6% to the overall structure of cancer cases. Bladder cancer ranks fourth (6.6% of new cancer cases in 2020), and kidney cancer ranks ninth (3.8%). Regarding male cancer mortality, prostate cancer remains the second leading cause (10.6% of deaths), while bladder cancer (5.8%) and kidney cancer (2.6%) are ranked fourth and ninth, respectively [3].

By 2020, in females, urologic cancer played a lesser role in the incidence of malignant diseases, as kidney cancer was ranked tenth, accounting for 2.4% of new female cancer cases. Regarding mortality, urologic cancers are ranked low and classified as “other” [3].

## 2. Methods

In this narrative review, we present and discuss the national and foreign literature that is considered relevant by the authors in the context of urogenital cancer epidemiology in Poland. A non-systematic literature search was performed using the PubMed and Google Scholar databases, using the following keywords: epidemiology, mortality, incidence, survival, Poland, urogenital cancer, prostate cancer, bladder cancer, kidney cancer, testicular cancer, and penile cancer.

Most of the incidence data comes from the Polish National Cancer Registry (PNCR). In Poland, the PNCR is the oldest structured database, established in 1952. Mortality data came from Statistics Poland. In Poland, a uniform protocol is applied throughout the country, which allows the same rules for cancer registration to be maintained in the whole country. Cancer registration in PNCR is mandatory, which ensures a high level of completeness of the data. PNCR registry data are partly based on clinical reports made by a wide range of clinicians, with an inherent risk of some coding errors.

Some data come from the National Health Fund. The National Health Fund began operating in 2003. The National Health Fund database applies only and exclusively to people diagnosed and treated within the public health protection system.

## 3. Results

### 3.1. Prostate Cancer

According to 2020 data, prostate cancer accounts for 20% of all male cancer cases in Poland [3]. The dynamics of prostate cancer incidence in Poland have accelerated between the years 2000 and 2015—the annual percentage change (APC) was 7.1% (95% CI 6.7 to 7.5) [3,4]. In the period 2015–2019, before the COVID-19 pandemic occurred, prostate cancer incidence increased, then decreased in 2020, and in 2021, a disproportionate increase in incidence rates was observed (data from the National Health Fund of Poland) [5]. The estimated incidence of prostate cancer was 22,344 men in 2025 compared to 12,162 in 2013 [6]. The absence of early detection programs for prostate cancer in Poland during the analyzed period highlights a significant gap in preventive healthcare measures. However, according to Chandran et al., the Prostate Cancer Awareness and Initiative for Screening Europe (PRAISE-U) pilot study is scheduled to commence in the third quarter of 2024 for a duration of 12 months in five European sites, including Lower Silesia, Poland. The detection process involves PSA evaluation, followed by risk assessment before and potentially after magnetic resonance imaging, ultimately leading to a biopsy decision [7]. If such screening programs were to be implemented nationwide in Poland, it would likely lead to a significant increase in prostate cancer incidence rates, particularly in the initial years following implementation. This surge would be attributed to the detection of previously undiagnosed cases, including those in early stages. Consequently, the current predictions for prostate cancer incidence in Poland may underestimate the potential impact of widespread screening, emphasizing the need for healthcare systems to prepare for a possible increase in diagnostic and treatment demands.

The analysis of PCNCR data showed a slight increase in prostate cancer mortality since 1990, which continued until the end of the twentieth century, followed by a stabilization period. Since 2010, a renewed increase has been observed, which remained evident until 2020 [8]. Since the 2000s, we have observed a slight decrease in age-related prostate cancer mortality; however, this dynamic is completely eliminated by population ageing processes (even though men constitute only 28% of the population over 85 years of age) [9]. In the 2020 PNCR report, prostate cancer was the second most common cause of cancer mortality in men (10.6%), followed by lung cancer (26.1%) [8]. Wong et al. classified Poland into countries with increasing incidence and decreasing mortality due to PCa using data from the Cancer Incidence in Five Continents database and the WHO mortality database for 2012 [10]. The annual number of registered deaths due to prostate cancer has increased from 4440 in 2014 to 6550 in 2030 [9]. According to the age-period-cohort model, there is an expectation of growth in the number of deaths from prostate cancer registered in Poland in the years 2018–2040, most rapidly among all cancer sites [9].

In Poland, the relation of standardized death rates (SDR) in men with primary education to SDR in men with higher education was 4.8 in 2011 [11], and mortality differences were marked with a disadvantage for urban residents compared to rural residents [11]. Regarding the standard expected years of life lost per death per person (SEYLL), PCa was ranked 16th in the male group compared to 20 diseases, and was the most common cause of death in Poland in 2011 [11].

Four studies [6,8,12,13] have addressed the relative survival of patients with prostate cancer. The 5-year relative survival rate for patients diagnosed in the years 1999–2010 increased from 61% (1999–2001) [8] to 70.6% in the period 2000–2004 and 76.6% (2005–2009) [13]. In the years 2010–2014, age-standardized 5-year prostate cancer survival rate was 81.8% [13]. The lowest survival rate was observed in the Opolskie voivodship (72.3%) [6]. In the years 2000–2002, 1-year relative survival rates for prostate cancer patients were 84.1%, and in the years 2003–2005, 87.8% (analysis of PCNCR data) [12]. In the CONCORD-3 analysis, they observed over 90% a 5-year survival rate in most European countries—Poland was among five countries where the survival rate was <80% [6]. According to some Polish authors, prostate cancer’s 5-year relative survival rate is lower than the European average due to the advanced stage at the beginning of treatment and possibly delayed treatment initiation [14].

Prostate cancer is rarely diagnosed before the age of 50 (less than 1% of cases) [12]. In Poland, 90% of new cases and 97% of deaths occur after 60 years of age [15]. Due to the aging of the population, prostate cancer is expected to become an increasingly significant problem. In 2018, 14.1% of Polish men were over 65 years old; by 2035, this percentage is projected to reach 26% [15].

Understanding the risk factors for prostate cancer is very important in targeting primary and secondary prevention [16]. The risk of prostate cancer diagnosis increases with age, along with its time-dependent relationship, which is well illustrated by incidence analysis stratified by birth cohorts. In the Polish population, the risk of prostate cancer diagnosis increases with each age group, with a higher risk in later-born cohorts. For example, men aged 60–64 who were born between 1955 and 1959 demonstrated a 10-fold higher risk of diagnosis than men the same age born in 1905–1909 [15].

Additional risk factors include the occurrence of the disease among close family members (diagnosis in three first-degree relatives or two of them aged <55 years), which increases the risk of incidence by a factor of 5 [17]. Some researchers have suggested that exposure to pesticides and chemicals used in agriculture may increase the risk of prostate cancer [17]. In Poland, a cross-sectional survey was carried out between 1 and 4 March 2024 in a nationwide sample of 2165 adults to assess the awareness of genitourinary cancer risk factors among adults. In total, 50% of the respondents indicated that family history and older age were the most recognizable risk factors for prostate cancer. Additionally, 34% were aware that chemical exposure increases the risk of prostate cancer [18].

In particular, several studies have excluded the influence of smoking and alcohol consumption and have not conclusively established the role of diet (for example, processed meat and milk consumption, inflammatory and hyperinsulinemic diets), hormonal exposure, and occupational exposure as risk factors for this disease [17].

There is also strong evidence that genetic factors such as germline mutations in DNA repair genes are associated with the risk of prostate cancer [17].

Some papers indicate metabolic syndrome, obesity, taller height, physical activity, Sjogren’s syndrome, periodontal disease, HPV, and marital status as risk factors for prostate cancer [16]. According to data from Statistics Poland [19] published every 5–6 years, in 2019, 56.6% of people over 15 years of age were overweight or obese.

The forecasts of the National Health Fund [20] for Poland show that in 2035, over 35% of adult men and over 25% of adult women will be obese.

A systematic review and random-effect meta-analysis found that circumcised men had a lower risk of prostate cancer [21]. Men with >21 ejaculations per month had a reduced risk of both overall and aggressive prostate cancer [22].

In a paper discussing an empirical model of prostate cancer treatment in Poland, attention was also drawn to the discrepancies between the PNCR and the National Health Fund data. The PNCR database is the only direct source of information about the cancer stage, but for some of the newly reported cases, there is no information about the disease stage because the method of entering data into the database does not force the person reporting the disease to fill out all fields of the reporting form. The PNCR database is struggling with the problems of non-registration of individual cases and incompleteness of reported data. In turn, the National Health Fund database applies only and exclusively to people diagnosed and treated within the public health protection system, and the period of its operation is much shorter than the period of operation of PNCR [23].

In recent years, the heterogeneous nature of prostate cancer has become a significant epidemiological, diagnostic, and therapeutic problem [12]. In Poland, there is not a prostate cancer screening program. The diagnostic evaluation of prostate cancer is based on individual opportunistic early detection depending on individual risk factors in well-informed men with a higher risk of prostate cancer. According to the European Association of Urology guidelines, which apply in Poland, it should include men from 50 years of age, men from 45 years of age and a family history of prostate cancer, men carrying BRCA2 mutations from 40 years of age, and men of African descent from 45 years of age [24]. Due to the uncertain benefits of screening, there is a risk of overdiagnosis and overtreatment for population-based screening programs like in colorectal, cervical, or breast cancer. The majority of screening tests include the PSA and digital rectal examination; however, the role of new imaging methods is growing. The 68Ga-PSMA PET/CT vs. multiparametric magnetic resonance imaging targeted biopsy in the diagnosis of clinically significant prostate cancer showed that 68Ga-PSMA PET/CT scans have good diagnostic accuracy (92% vs. 86.2%), which may change the guidelines in the future. Also, additional tools to PSA are needed to improve the diagnostic value of the marker. In Pepe et al.’s study using percent-free PSA thresholds of 15% (for PSA ≤ 2.5 ng/mL) and 20% (for PSA 2.6–4 ng/mL), prostate cancer was detected in 25.6% and 27.4% of cases, respectively. Importantly, the majority of these cancers were clinically significant, with organ-confined disease observed in 70.8% of patients undergoing radical prostatectomy. This highlights the value of these thresholds in identifying clinically relevant PCa in men with PSA ≤ 4 ng/mL, making them suitable candidates for curative nerve-sparing surgery [25]. A 2005 study in Sicily demonstrated the PSA test’s value in early detection and tailoring diagnosis timing. This approach considers both age and PSA levels to optimize early prostate cancer diagnosis [26].

Data on the stage of prostate cancer in Poland are fragmentary. Only data collected by PNCR in 2018 for 2010 and 2016 covered the entire population. In 2010, 68% of registered prostate cancer cases were local, 12% regional, and 20% distant. In 2016, the number of registered cases of prostate cancer with local advancement increased to 80%, with regional advancement dropped to 7%, and distant advancement to 13% [3].

In Table 1, we present epidemiological studies of prostate cancer in Poland.

### 3.2. Bladder Cancer

Each year, bladder cancer is diagnosed in more than 6000 residents of Poland [27]. It was ranked fourth in terms of male cancer incidence in Poland in 2020 [8] and 14th among women in 2013, although it remains the most common urinary malignancy among female patients [27].

Two of the analyzed studies [28,29] and PNCR data [8] consistently pointed to an increasing trend in the incidence of bladder cancer in Poland from 1980 to 2013. A plateau was observed between 2014 and 2019, followed by a temporary decrease in incidence in 2020 due to the COVID-19 pandemic. Differences in incidence were observed between urban and rural inhabitants and between particular voivodeships, with the elevated numbers reported for the voivodeship Łódź, most probably due to the textile dying industry in this area.

Bladder cancer mortality rates in Poland have increased steadily since the 1980s [28]. In the years 2000–2014, bladder cancer was the cause of death in 44,283 inhabitants of Poland [30]. In 2020, 3202 male deaths and 915 female deaths were reported [3]. In 2000, SEYLL for men who died due to bladder cancer was 18.8 years of life and for women 16.2 years, while in 2014, it was only 17 years for men and 15.7 years for women [30]. The number of life years lost due to bladder cancer also decreased, but mortality continued to increase, which could be the result of the aging Polish population [30].

Interestingly, bladder cancer is four times more common in men than in women [17]. Bladder cancer is one of the most prevalent cancers in older individuals, with 75% of cases diagnosed in patients over 65 years of age [17].

The 5-year age-standardized survival rates for men in Poland were 60.4% in 2000–2004, 63.7% in 2005–2009, and 63.3% in 2010–2014. For women, the rates during the same periods were 63.1%, 66.0% and 64.9%, respectively [8,13].

Bladder cancer is significantly linked to smoking. In Poland, 35.3% of the respondents indicated smoking as a risk factor for bladder cancer [18]. In addition, one-quarter of adults in Poland are smokers [31]. The connection between this cancer and occupational exposure (aluminum production, rubber production, painting, firefighting [32]), especially to aromatic amines in plants in the chemical industry, has been well documented. In total, 39.4% of respondents in Poland were aware that chemical exposure increases the risk of bladder cancer [18] and 51.9% that chronic bladder irritation and infections are responsible for this. In the Middle East, North Africa, India, and Australia, bladder cancer is also associated with exposure to infectious agents, such as Schistosoma haematobium [17].

Bladder cancer is one of the most prevalent cancers in older individuals, with 75% of cases diagnosed in patients over 65 years of age. Interestingly, this cancer is four times more common in men than in women and is more prevalent in Caucasians than in individuals of the black race [17].

A 2023 systemic review lists environmental exposures (X radiation or gamma radiation, and arsenic), medications (cyclophosphamide and chlornaphazine), and opium consumption as risk factors for bladder cancer [32].

Data on the stage of bladder cancer in Poland are fragmentary. Only data collected by PNCR in 2018 for 2010 and 2016 covered the entire population. In 2010, 75% of registered prostate cancer cases were local, 14% regional, and 11% distant. In 2016, the number of registered cases of bladder cancer with local advancement increased to 82%, regional advancement dropped to 8% and distant advancement to 10% [3].

Table 2 presents epidemiological studies of bladder cancer conducted in Poland.

### 3.3. Kidney Cancer

Kidney cancer (C64, malignant neoplasm of the kidney, except the renal pelvis) represents approximately 2–3% of all adult malignancies worldwide. The Polish population has an intermediate incidence rate of this cancer, with 2778 cases in men and 1788 cases in women in 2020 [3]. The collected data do not encompass cases of malignant neoplasm of the renal pelvis (C65) and malignant neoplasm of the ureter (C66) due to the uncommon nature of these diseases and the disparate treatment methods employed, which results in insufficient data. Further research should be conducted to investigate the prevalence of upper tract urothelial carcinoma (UTUC) in Poland.

According to the studies analyzed, in Poland, the incidence of kidney cancer in both sexes increased until the mid-1990s, followed by over a decade of stabilization [17,33], a slight decrease after 2017, and a significant decrease from 2019 (PNCR data).

Mortality, as with incidence, escalated until the mid-1990s. Since then, mortality trends in both sexes have stabilized [17,33].

In Poland, approximately 50% of cases are observed in the oldest age group (65 years). Among individuals of both sexes, the majority of deaths occur after the age of 65 years (60% in males and 70% in females). Incidence and mortality due to kidney cancer are linearly correlated with age [3].

Two studies [13,17] and PNCR data addressed the issue of 5-year relative survival rates in Poland. In the years 2000–2002, the 5-year relative survival rate was reported to be approximately 50% in both sexes [8,13,17]. In the years 2000–2015, the age-standardized 5-year survival rate for kidney cancer increased across both sexes. Among women in the years 2000–2004, it was 59.3%; in the years 2005–2009, it was 65.6%; and in the years 2010–2014, it was 70.6%. During the same observation period, the survival rates of the men were 54.3%, 58.8%, and 63.9%, respectively. Survival rates vary across voivodships [8,13,17].

The origins of kidney cancer remain unclear. Various factors have been associated with increased risk, including smoking (with an attributable risk of approximately 20% in men and 10% in women), obesity, a high-protein diet, prolonged use of pain relievers, hypertension, and extended use of diuretics [17].

The strongest risk factors of renal cell carcinoma are age and sex [34].

In total, 40.6% of the Poles who responded recognized smoking as a risk factor for kidney cancer, 50.1% exposure to chemicals, and less than 30% chronic use of analgesics or long-term dialysis treatment [18].

Data on the stage of kidney cancer in Poland are fragmentary. Only data collected by PNCR in 2018 for 2010 and 2016 covered the entire population. In 2010, 63% of registered kidney cancer cases were local, 11% regional, and 26% distant. In 2016, the number of registered cases of kidney cancer with local advancement increased to 70%, while regional advancement dropped to 7% and distant advancement to 23% [3].

### 3.4. Testicular Cancer

Testicular cancer constitutes the lowest proportion of urogenital malignancies. In 2020, 1156 men were diagnosed with this type of cancer, 136 of whom died [3].

The incidence rates in Poland are among the lowest in Europe [35]. The incidence of this cancer is constantly increasing (although there was a drop in 2020 due to the COVID-19 pandemic) [36].

The increase in testicular cancer morbidity is not steady in all age groups, which pertains to young people to a greater extent [35]. Despite its low prevalence, accounting for only 1.6% of malignant tumors in men, it is the most common cancer diagnosed in young men aged 20–44 years [36]. In this age group, testicular cancer represented 26% of new cancer cases and caused 7% of cancer-related deaths in 2020 [17,33]. The peak risk of diagnosis falls between the ages of 25 and 30 years, and approximately 70% of cases occur before the age of 40 [36].

However, the distribution of testicular cancer by age is characterized by two distinct peaks, with the highest incidence occurring between 20 and 34 years of age and a second increase observed in the age range of 60 to 70 years [17].

In 1980, Poland reported the third-highest number of cancer-related deaths among young men, with testicular cancer being the leading cause. It was estimated that approximately 300 new cases of testicular cancer occur annually in Poland, resulting in an incidence rate of 1.6 per 100,000 men [8]. The number of deaths due to testicular cancer has steadily increased annually since the 1980s (PCNCR data). The trend of testicular cancer mortality was slightly higher than the average for the EU countries [35]. Testicular cancer represents an epidemiological problem raised in the Polish medical literature. Five articles discussing data from more than 10 years ago were identified [35,36,37,38,39]. In the Lancet, two articles were published in English on the epidemiology of testicular cancer in Poland [38,39]. It was observed that the increasing time trend in testicular cancer incidence in the years 1963–1988 was similar to that observed in Western Europe. In mortality, a decline started in 1981; therefore, the trend of mortality over time was similar to that in Western Europe, with a delay of approximately 10 years [39].

A 1990s study on trends in mortality related to urological causes in the years 1970–1985 highlighted testicular cancer as the only malignant disease characterized by an increase in incidence and a simultaneous decrease in mortality during the analyzed period [40].

Based on observations of over 500 patients treated at the M. Sklodowska-Curie Memorial Cancer Center and Institute of Oncology in the years 1979–1988, the probability of 5-year survival for patients with testicular cancer was estimated to be 80% [38]. Testicular cancer is characterized by the highest survival rates among males diagnosed with a malignant disease: in the years 2000–2004, it was 85.3%, in 2005–2009, 86.2%, and in the period 2010–2014, 89.5%, with exact values varying between different voivodships [8,13].

The majority of testicular tumors (approximately 95%) are germ cell tumors, which are typically divided into seminomas (approximately 50%) and non-seminomas (>40% of testicular cancer cases in European populations). The highest incidence of seminoma occurs on average 10 years later (30–45 years of age) than that of non-seminoma (20–40 years of age) [17].

These tumors typically occur more frequently in the Northern Hemisphere, particularly among individuals of Caucasian descent. On the contrary, they are less common in Asian and African countries. Among the Caucasian population, the incidence of these cancers increases by 3 to 6% annually, suggesting significant changes in exposure to risk factors. Patients with testicular cancer are more likely to have cryptorchidism (attributable risk estimated at 10%), inguinal hernia, hydrocele, testicular atrophy, and infertility than the general population [17].

Factors associated with an increased risk of testicular germ cell tumors include Klinefelter syndrome, a history of testicular cancer in first-degree relatives (father or brother), and a history of germ cell tumors in the opposite testicle. The risk of developing second testicular cancer is approximately 5%. Unfavorable environmental changes appear to play an important role, as there has been a significant increase in the incidence of this cancer in recent decades [8].

Risk factors also include age, height (there are several studies confirming a directly proportional relationship between height and the risk of testicular malignancy), fertility disorders, hypocrisy, Down’s syndrome, HIV [41], exposure to pesticides, use of recreational drugs, and, in particular, cannabis [42].

However, older ages of mothers at conception were associated with a reduced risk of testicular cancer in addition to a relatively lower risk in men who had been breastfed for 6 months or more [42]. A meta-analysis by Maule et al. involving 12 studies showed a protective role of late puberty in testicular cancer [43].

The utility of screening for the early detection of testicular cancer has not been proven. Self-examination of the testes allows for early disease detection but does not reduce disease-specific mortality [36].

In Table 3, we present epidemiological studies of testicular cancer in Poland.

### 3.5. Penile Cancer

Penile cancer is a rare malignancy, accounting for 0.3% of new cancer cases among men and is responsible for 0.3% of men’s cancer-related deaths [44].

The main risk factors for penile cancer include phimosis, chronic inflammation of the glans penis and foreskin, UVA phototherapy, phototherapy with the use of psolarenes, smoking, HPV infection, low socioeconomic status, living in rural areas, early age of sexual initiation, a high number of sexual partners, and unmarried men. Prevention methods include HPV vaccination, the use of condoms to reduce HPV infections, self-examination as part of daily genital toileting, smoking cessation, treatment of chronic penile inflammation, reduction of the use of photochemotherapy and personal hygiene [45].

Most Polish articles on penile cancer are limited to case reports or address the quality of life of patients with this disease [46]. In an article published in the 1990s discussing mortality trends from urological causes, it was mentioned that an increase was in the analyzed period for kidney, bladder, prostate, and testicular cancers; however, the mortality rate due to penile cancer did not show a noticeable change [40].

In the Polish literature, we identified only two studies that strictly addressed the epidemiology of penile cancer [44,45]. The article, not published in a scientific journal but available online on the website of the Mazovian Branch of the PUA, presented data for 2012. According to this article, 218 men were diagnosed with the disease in Poland in 2012. From 1990 to 2012, no statistically significant changes in incidence were observed [44].

The risk of developing the disease increases with age and peaks between 50 and 70 years, regardless of race. Approximately 90% of new cases occur after the age of 50 years, and the 5-year survival rate in Poland in 2012 was 63.6% [44].

In the 40+ men group, in the years 1990–2020, intermediate incidence values (ASIR 1.88–2.61) and intermediate mortality values (ASMR 0.74–0.99) were observed for Poland. The incidence and mortality of penile cancer in the 40+ and 0+ men groups increased over the years analyzed [45].

The 5-year relative survival of European penile cancer patients diagnosed in the period 1990–2007 from the EUNICE (European Network for Indicators on Cancer) database was 67% [47]. In 2020, survival rates in Poland deteriorated by 60% [45].

In Table 4, we present the penile cancer epidemiological studies in Poland.

## 4. Discussion

The number of publications on the epidemiology of uro-oncology in Poland is very small, and the data analyzed came from the period 10–20 years ago and are not updated, except for penile cancer. Actual data can be found mainly in PNCR bulletins.

Most of the articles included in our review were published in Polish. Prostate cancer is the most discussed cancer, but there are no separate publications on kidney cancer.

The epidemiological data on urogenital cancer presented in this article should be approached with skepticism, given that the breakthrough in improving the completeness of the data in the PNCR occurred in the mid-1990s. The COVID-19 pandemic further distorted the observed trends due to limited contact with healthcare and the transformation of many centers into single-purpose hospitals, which disrupted the process of cancer diagnosis and treatment in most European countries and the USA. There is also no information from the National Health Fund on the treatment of urological cancers during the pandemic period.

We believe that new articles should be written on epidemiology in uro-oncology in Poland. Only in this way can we have information about changes taking place in morbidity and mortality in relation to implemented treatment methods, health awareness, and screening.

Furthermore, we are of the opinion that the increasing number of cancer patients constitutes a challenge in the diagnosis and treatment, especially in healthcare with limited resources. The uro-oncologist should focus on cancer prevention and spread knowledge about cancer-related risk factors listed and explained in the European Code against Cancer.

More educational campaigns should be implemented on risk factors for urogenital cancers in Poland—it is very important to target primary and secondary prevention. Currently, in Poland, educational campaigns on urogenital cancers are mainly limited to those dedicated to prostate and testicular cancer during the annual “Movember” event and Urology Week.

## 5. Limitations

Given the limited number of articles on this subject, we opted to incorporate articles from a historical period in our analysis. To this end, we sought all available data, including non-peer-reviewed and non-indexed sources. We acknowledge the limitations of these data sources, but due to the dearth of indexed journal data, we analyzed all available publications. This is the first large-scale epidemiological study. In subsequent analyses, it would be advisable to focus on indexed sources, which will now be feasible due to contemporary cancer registration systems.

Prostate cancer epidemiological studies coming from Poland include only eight publications that are important from the authors’ point of view. In these works, incidence and mortality are analyzed until 2015.

Bladder cancer epidemiological studies coming from Poland include three publications that are important from the authors’ point of view. In these works, incidence, mortality, and survival are analyzed until 2014.

There are no separate Polish studies devoted to the epidemiology of kidney cancer.

Epidemiological studies of testicular cancer from Poland include five papers. The data analyzed there cover the period until 2014.

## 6. Strengths

Data on urogenital cancer epidemiology in Poland can be found on the IARC website (WHO agency) [48]; however, these data are only estimates. Poland has submitted comments several times regarding the methodology for estimating the number of cases in Poland (the last contact on this matter was with Mr. Freddy Bray in November 2024).

The data for Poland presented by the IARC are estimated on a sub-population basis. Based on the mortality rate, the incidence is estimated for six voivodships, except for prostate cancer, where the estimate was prepared on the basis of only two voivodships. GCO data on incidence ended in 2017. Another disadvantage of the GCO website is that it uses standardization for the standard world population, which differs significantly from the values observed in Europe. For example, the value of the prostate cancer incidence rate in 2017 was estimated at 58.6, while according to PNCR data, this value is 50.02, and the crude rate is 89.96, which means that the value reported in GCO is 35% lower than the real threat of this cancer to the Polish population.

Mortality is also estimated. In-depth studies conducted at PNCR showed that the data used for the estimations differ from the official data from Statistics Poland. Survival rates are also underestimated.

We believe that the reliable and factual presentation of the epidemiological picture of urogenital cancers in Poland contained in our work is valuable for both Polish and foreign readers.

## 7. Conclusions

The number of publications on urogenital cancer epidemiology in Poland is small. The number of such publications worldwide is also small.

Polish studies lack data from the National Health Fund and the National Consultant in Urology.

Data from the COVID-19 period are distorted due to the reduction in the number of screening tests performed, limited contact with health care, and the transformation of many hospitals into single-name hospitals.

Studies on the epidemiology of urological cancers should be conducted to update the data and organize the epidemiological knowledge.

## Figures and Tables

**Table 1:** Prostate cancer epidemiological studies coming out of Poland.

Author	Title	Journal/Book	Main topic	Reference
Dobruch et al.	Epidemiologia raka gruczołu krokowego: zmiany obserwowane w Polsce w latach 1991–2000	Urol.Pol. 2005; 58; 1	prostate cancer incidence and mortality 1991–2000	[was not cited; duplicated PNCR data]
Sosnowski et al.	Rak gruczołu krokowego—istotny problem epidemiologiczny i kliniczny we współczesnej medycynie	Medycyna po Dyplomie. 2014	prostate cancer incidence and mortality 1980–2011	[12]
Didkowska et al.	Programy wczesnego wykrywania raka stercza—krytyczna analiza okiem epidemiologa	W: Wiechno P. Rak stercza. Współczesne spojrzenie. PZWL, 2021; str. 22–32.	prostate cancer incidence in age groups according to birth years	[15]
Rachtan et al.	Zachorowalność na nowotwory złośliwe gruczołu krokowego w województwie małopolskim w latach 1999–2001	Urol.Pol. 2005; 58; 3.	prostate cancer incidence in Małopolskie voivodeship 199–2001	[was not cited; duplicated PNCR data at regional level]
Osowiecka et al.	The Waiting Time of Prostate Cancer Patients in Poland.	Int J Environ Res Public Health. 2019; 16(3): 342.	five-year prostate cancer relative survival compared to European average	[14]
Pikala et al.	Epidemiology of Mortality Due to Prostate Cancer in Poland, 2000–2015	Int J Environ Res Public Health. 2019; 16(16): 2881.	prostate cancer incidence and mortality in Poland in years 2000–2015	[4]
Czaderny K.	High prostate cancer mortality in Poland. A spatial, temporal and structural analysis	Przegl Epidemiol. 2018; 72(2): 235–246.	prostate cancer mortality (1980–2014) trends and prognosis for years 2018–2040	[9]
Gliniewicz et al.	Survival in men diagnosed with prostate cancer in Poland in the years 2000–2014 compared to European Countries based on Concord-3.	Rocz Panstw Zakl Hig. 2020; 71(4): 445–453.	five-year prostate cancer survival in Poland (2000–2014)	[6]

**Table 2:** Bladder cancer epidemiological studies coming out of Poland.

Author	Title	Journal/Book	Main Topic Reference
Jabłonowski Z.	Rak pęcherza moczowego– epidemiologia, diagnostyka i leczenie w XXI wieku	Folia Medica Lodziensia. 2013; 40; 1: 31–52	bladder cancer epidemiology, diagnosis, treatment [27]
Szkodny et al.	Naciekający rak pęcherza moczowego (T2 T3 T4)	Urol.Pol. 1989; 42; 3.	bladder cancer mortality 1970–1985, incidence 1952–1982, treatment 1985. [28]
Jobczyk et al.	Years of life lost due to bladder cancer among the inhabitants of Poland in the years 2000 to 2014.	Cent European J Urol. 2017; 70(4): 338–343.	first study to assess [30] the burden of bladder cancer in Polish patients, expressed as years of life lost

**Table 3:** Testicular cancer epidemiological studies coming out of Poland.

Author	Title	Journal/Book	Main Topic	Reference
Stawińska-Witoszyńska et al.	Malignant testicular tumour incidence and mortality trends.	Contemp Oncol (Pozn). 2016; 20(1): 58–62.	comparative analysis of testicular cancer incidence and mortality trends among men in Poland and in Wielkopolska Province	[35]
Zatoński et al.	Testicular cancer in Poland.	Lancet. 1990; 336(8708): 183.	Incidence and mortality trends in Poland (1963–1988) compared to trends in Europe	[39]
Madej G.	Treatment of testicular cancer in Poland.	Lancet. 1990; 336(8712): 440	Incidence and mortality trends in Poland 1979–1988. Probable 5-year survival	[38]
Adamkiewicz K.	Złośliwe nowotwory jądra i ich leczenie w Klinice Urologii AM w Gdańsku w latach 1975–1985.	Urol.Pol. 1987; 40; 1	Incidence and mortality of testicular cancer in Poland in 1980	[37]
Sugajska et al.	Ryzyko wystąpienia raka jądra w świadomości młodych mężczyzn.	NOWOTWORY. 2020; 5; 6: 321–326.	Testicular cancer incidence and age	[36]

**Table 4:** Penile cancer epidemiological studies coming out of Poland.

Author	Title	Journal/Book	Main Topic	Reference
Sobieszczuk M.	Rak prącia—Epidemiologia i czynniki ryzyka	http://www.ptumazowsze.org.pl/rak-pracia-epidemiologia.html (accessed on 28 March 2023).	penile cancer epidemiology and risk factors	[44]
Wnętrzak et al.	Epidemiology of penile cancer in Poland compared to other European countries.	Cancer Med. 2024; 13(16): e70092.	penile cancer epidemiology in Poland compared to other European countries	[45]
